# Testicular Neoplasms and Other Abnormalities in Common Carp *Cyprinus carpio* from the Lower Colorado River, United States

**DOI:** 10.3390/ani15192887

**Published:** 2025-10-02

**Authors:** Vicki S. Blazer, Steven L. Goodbred, Heather L. Walsh, Dylan Wichman, Darren Johnson, Reynaldo Patiño

**Affiliations:** 1 U.S. Geological Survey, Eastern Ecological Science Center—Leetown Research Laboratory, Kearneysville, WV 25430, USA; hwalsh@usgs.gov (H.L.W.); dwichman@usgs.gov (D.W.); 2 U.S. Geological Survey, California Water Science Center, Sacramento, CA 95819, USA; sgoodbred@usgs.gov; 3 Contractor to the U.S. Geological Survey, Wetland and Aquatic Research Center, Lafayette, LA 70506, USA; johnsond@contractor.usgs.gov; 4 U.S. Geological Survey, Texas Cooperative Fish and Wildlife Unit, Texas Tech University, Lubbock, TX 79409, USA; reynaldo.patino@ttu.edu

**Keywords:** common carp, testicular neoplasms, testicular macrophage aggregates, Sertoli cell proliferation, Colorado River

## Abstract

Abnormalities were observed in the testes of common carp *Cyprinus carpio* collected from Willow Beach, Arizona, USA, a site on the lower Colorado River, downstream of Lake Mead and Hoover Dam. Testicular tissue collected from this site in 2003 exhibited numerous large, pigmented macrophage aggregates (MAs) and a novel, previously undescribed hypertrophy and proliferation of putative Sertoli cells. In testes samples collected in 2007, numerous testicular MA, testicular oocytes, and proliferations of Sertoli cells were observed. Three carp collected in 2007 also had raised nodules within the testes, and, microscopically, seminoma, spermatogenic seminoma, and mixed stromal cell–germ cell neoplasms were diagnosed. Several risk factors for these adverse effects were identified. Carp collected at this site in 2003 ranged in age from 35 to 54 years and had the oldest mean age of the thirteen sites sampled within the Colorado River basin. This site also has an unusual thermal regime when compared to other sites studied in Lake Mead and upstream sites, in that temperatures varied little over the seasons (amplitude around 1.5 °C) and barely reached 15 °C. Additionally, carp from this site had the highest total polychlorinated biphenyl (PCB) body burden. Hence, advanced age, low water temperature, and exposure to PCBs and other environmental contaminants may contribute to the observed abnormalities, highlighting the complex environmental factors initiating pre-neoplastic and neoplastic changes in wild carp.

## Simple Summary

Common carp are used as an indicator species in the Colorado River and elsewhere for fish health and reproductive studies. Carp were collected at one site, Willow Beach, downstream of Lake Mead and Hoover Dam, at multiple times since 2003. Tumors, including seminoma, spermatogenic seminoma, and mixed germ cell–stromal neoplasms, were observed. Other testicular abnormalities, including Sertoli cells, pigmented macrophage aggregates, and intersex (testicular oocytes), were also found. Carp collected at this site in 2003 ranged in age from 35 to 54 years. They also had the highest total PCB (polychlorinated biphenyls) body burden compared to other sites within the Colorado River watershed. Additionally, this site has an unusual thermal regime when compared to other sites studied in Lake Mead and upstream sites, in that temperatures varied little over the seasons (amplitude around 1.5 °C) and barely reached 15 °C, which is considered the lowest temperature for spawning.

## 1. Introduction

Testicular tumors and other male reproductive abnormalities have been observed in many fish species worldwide. Intersex, as evidenced by oocytes (most often immature) within the testes of gonochoristic fishes, is perhaps the most common abnormality in terms of the number of species affected and prevalence within certain populations [1,2,3,4,5]. Although intersex has been observed in common carp *Cyprinus carpio* (hereafter carp), it is less commonly observed than in certain other species. For example, of the 216 testes examined microscopically from 25 sites throughout the United States, only one (0.46%) had testicular oocytes [6]. In another study, no adult male carp, of 774 examined microscopically, collected in nine river systems throughout the United States, were observed to have testicular oocytes, and one female carp (of 798) was found to have foci of spermatogenic cells. Conversely, 44% of largemouth bass *Micropterus salmoides* and smallmouth bass *M. dolomieu* males were intersex in the same study [7]. In the Illinois River, 4% of male carp were identified as intersex, compared to 41% of male largemouth bass [8]. Intersex male carp (19%) were reported from a site in Spain known to be contaminated with estrogenic compounds [9].

Testicular neoplasms have been reported from carp and carp–goldfish *Carassius auratus* hybrids. Commonly, these have been case reports of individual aquarium fish such as the ornamental koi carp [10,11,12,13], or single wild carp [14,15]. However, in the Great Lakes drainage in the United States and Canada, two types of testicular tumors have been described in a high percentage of carp–goldfish hybrids. One type was primarily a Sertoli cell neoplasm, and the second type contained proliferations of Sertoli cells and spermatogonia. Presence of immature oocytes within testes was also observed [16]. Although contaminant-related site differences were not found for the hybrid tumors evaluated in 1981–1982 [17], site differences in the prevalence of hybrid gonadal tumors were suggested to relate to environmental contaminants in the Wellman River, New York [18]. In a reservoir in Spain, carp–goldfish hybrids were described with a variety of testicular tumors, including seminoma, leiomyoma, Sertoli cell, and spermatocytic seminoma [19].

**Figure 1:**
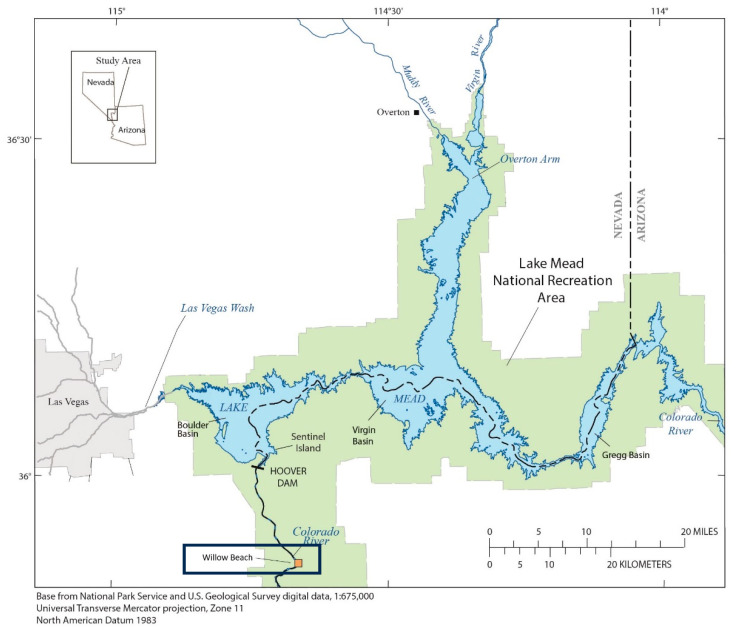
Willow Beach site (within the box) downstream of Lake Mead National Recreational Area and the Hoover Dam on the lower Colorado River.

Studies involving carp have occurred within Lake Mead and at downstream sites in the lower Colorado River Basin for many years. One of the sites at which carp have been collected is Willow Beach (WB), Arizona, approximately 20 river kilometers downstream from Hoover Dam (Figure 1).

This site receives water from the Las Vegas Bay in Lake Mead and the upstream drainage of the Colorado River. Carp were collected at WB and three sites within Lake Mead and its watershed every four months from March 2007 to March 2008 to assess contaminant body burdens and biomarkers of male reproductive condition [20]. Carp had been sampled from this site earlier during the Colorado River study of the U.S. Geological Survey’s Biomonitoring of Environmental Status and Trends (BEST) program in 2003 [21] and again during fish health assessments in 2010 [22]. The 2007 study focused on physiological (plasma) and organ (gonadosomatic index) biomarkers. However, the observation of visibly abnormal testes led to the preservation of a subsample of visibly abnormal and normal testes. This allowed us to perform histopathological analyses and describe the testicular tumors and other abnormalities in the current study. Additionally, utilizing archived slides and data from the 2003 study, we were able to investigate potential risk factors and determine that although the testicular tumors were not present in this earlier sampling period, some of the non-neoplastic abnormalities were present.

## 2. Methods

During the collection of samples for the BEST program in 2003, testes from nine male carp were preserved from the WB site on the Lower Colorado River in late September and processed for microscopic analysis as previously described [21]. Testes were collected from five carp at the same site in November 2007 [23] and preserved in 10% neutral buffered formalin. Pieces of preserved gonad from several areas along the testes were placed in cassettes, routinely processed for histology, embedded in paraffin, sectioned at 5–7 µm, and stained with hematoxylin and eosin [24]. The length, age, and gonad stage of these carp are presented in Table 1.

**Figure 2:**
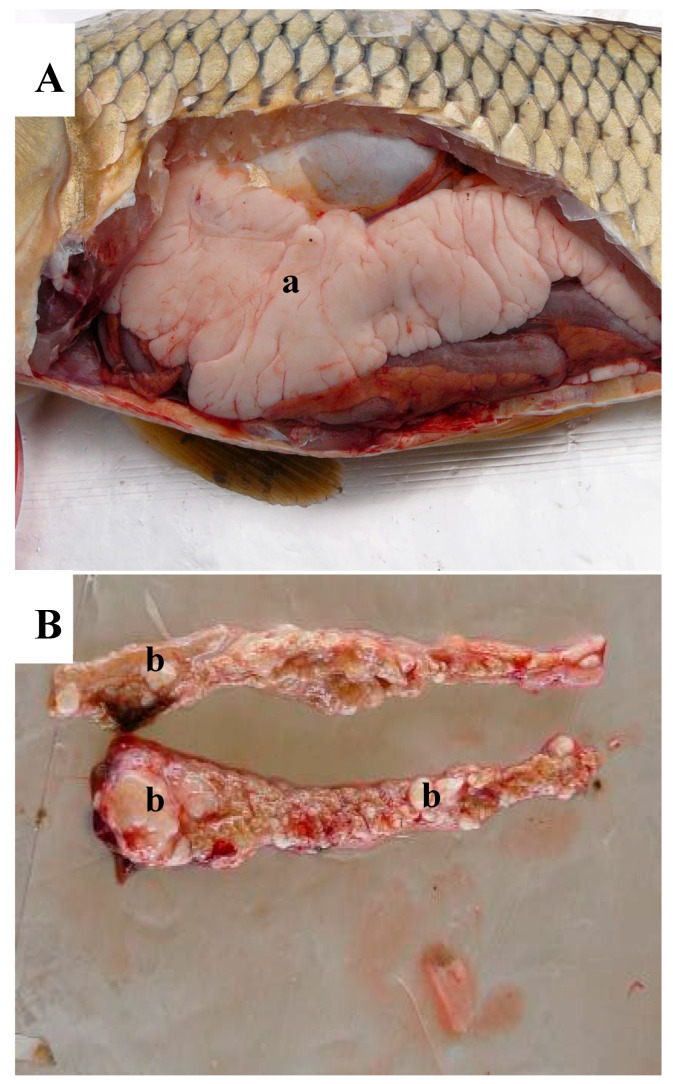
(**A**) Normal, mature testis (a) within the abdominal cavity of a common carp collected at Willow Beach, Arizona, 2003. (**B**) Abnormal testis from a common carp collected at Willow Beach, 2007. Gonads are small with numerous nodules of varying sizes throughout the tissue (b).

Two of the carp (WBCC-52 and 66) collected in 2007 showed no visually apparent abnormalities and were selected randomly for comparison with three carp (WBCC-46, 50, and 57) having abnormal-appearing testes. The abnormal testes were smaller than normal testis (Figure 2A) and had raised nodules. Carp WBCC-46 had large, raised nodules (Figure 2B), whereas carp WBCC-50 and WBCC-57 had smaller raised nodules.

## 3. Results

**Figure 3:**
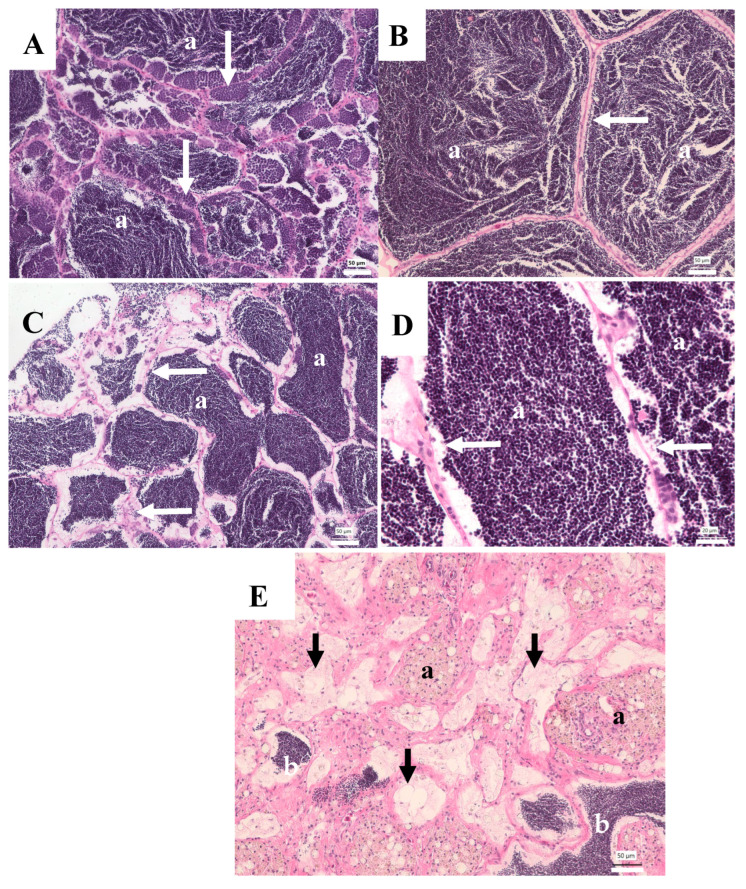
Testicular tissue of common carp collected from the Willow Beach site in 2003. (**A**) Normal stage 3 testis with tubules containing mature sperm (a) lined by spermatogonia, spermatocytes, and spermatids (arrows). Scale bar equals 50 µm. (**B**) Normal stage 4 testis tubules have a thin germinal epithelial (white arrow) and are filled with mature sperm (a). Scale bar equals 50 µm. (**C**) Stage 4 testis with abnormal germinal epithelium (arrows) of tubules filled with sperm (a). Scale bar equals 50 µm. (**D**) Higher magnification of C illustrating the highly vacuolated Sertoli cells lining the germinal epithelium (arrows). Scale bar equals 20 µm. (**E**) Abnormal testis with tubules lined by hypertrophied, vacuolated Sertoli cells (black arrows), large macrophage aggregates (a), and a few tubules containing sperm (b). Scale bar equals 50 µm. H&E stain.

Of the nine male carp collected in 2003 from the WB site, three testes were normal and four had moderate to extensive pigmented macrophage aggregates (MAs), but no other abnormalities (Table 1). The MAs contain yellowish-brown pigment consistent with ceroid-lipofuscin granules. Normal carp testis is composed of convoluted seminiferous tubules, interlobular interstitial cells, and blood vessels. In stage 3 (developing) testes, spermatogonia are abundant in the germinal epithelium, and, as development proceeds, cysts of spermatocytes and spermatids are observed, and many tubules contain sperm (Figure 3A). In stage 4 testes, the tubules are large with few visible spermatogonia, spermatocytes, and spermatids, and lumens are filled with sperm (Figure 3B). Both stages were observed in carp collected in 2003 (Table 1). The two other carp had testes containing abnormalities of the germinal epithelium as well as moderate to extensive MA. One had focal areas of hypertrophied cells believed to be Sertoli cells, lining the tubular epithelium (Figure 3C,D). These cells were highly vacuolated with a pale, granular appearance and small nuclei. Some cells appear to contain intracytoplasmic sperm. The testis of the other carp had areas with few normal tubules; some contained sperm, but many had empty lumens, lined with a proliferation of putative Sertoli cells. Many testicular MAs were noted in these areas (Figure 3E).

**Figure 4:**
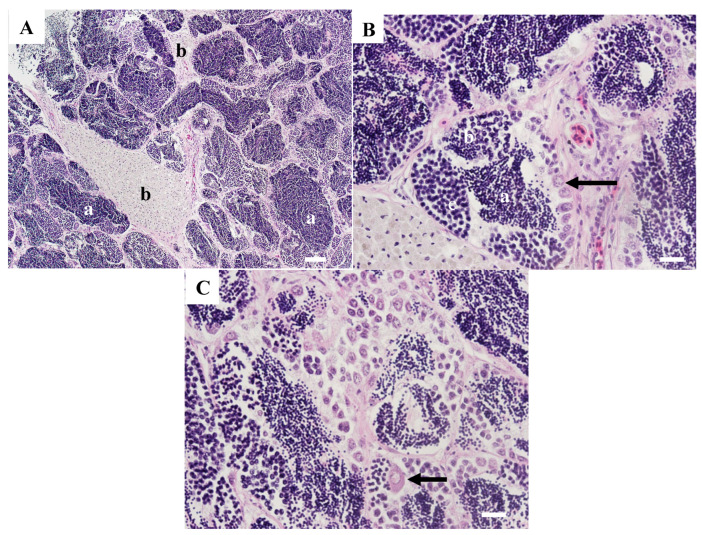
Testicular tissue from common carp WWCC-52 collected at the Willow Beach site in 2007. (**A**) Area of the testis with tubules containing sperm (a) and pigmented macrophage aggregates (b). Scale bar equals 50 µm. (**B**) Tubule containing sperm (a), spermatids (b), spermatocytes (c), and spermatogonia (black arrow). Scale bar equals 20 µm. (**C**) An immature oocyte (arrow) within a testicular cyst. Scale bar equals 20 µm. H&E stain.

Testicular tissue from two carp (WBCC-52 and 66) collected in 2007 had no grossly visible abnormalities; however, microscopically, abnormalities were present. WBCC-52 had primarily normal tubules filled with sperm (Figure 4A); however, there were large MAs present. In some tubules, spermatogonia, spermatocytes, spermatids, and sperm were evident (Figure 4B), and a few immature oocytes were identified (Figure 4C).

**Figure 5:**
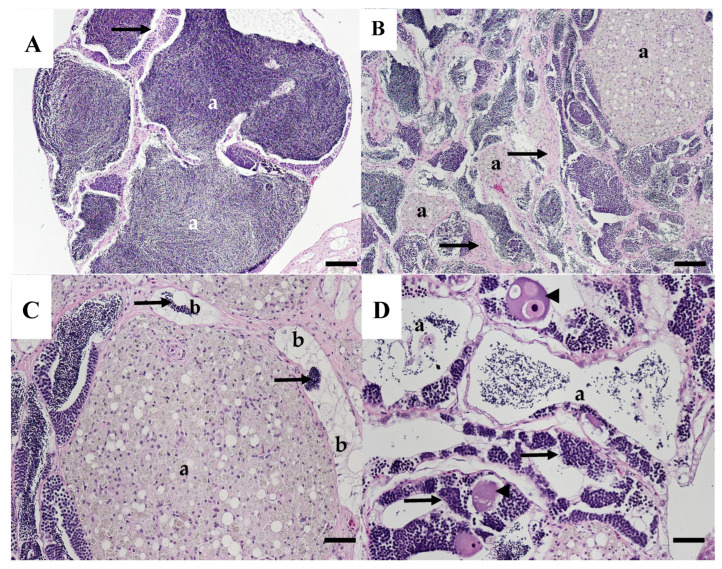
Microscopic appearance of testicular tissue from common carp WBCC-66 collected at the Willow Beach site in 2007. (**A**) Section of large tubules with sperm (a) and germinal epithelium containing spermatids and spermatocytes (arrow). Scale bar equals 100 µm. (**B**) Area of the testicular tissue with multiple, large, pigmented macrophage aggregates (a) and fibrotic connective tissue septae (arrows). Scale bar equals 100 µm. (**C**) Section with large aggregate macrophage aggregate (a) and tubules lined with large clear cells (b) and containing foci of possible sperm (arrows). Scale bar equals 50 µm. (**D**) Section with tubules containing a few sperm (a) and lined by germinal epithelium with spermatocytes, spermatids (arrows), and containing large, unidentified cells (arrowheads). Scale bar equals 50 µm. H&E stain.

Areas within the testis of WBCC-66 had large, sperm-filled tubules (Figure 5A), while other areas had multiple, large MAs and increased fibrous tissue (Figure 5B). Some tubules had normal germinal epithelium while others were lined with hypertrophied, vacuolated putative Sertoli cells, some of which had sperm in the lumen (Figure 5C). Some tubules with sperm in the lumens were lined with germinal epithelium containing spermatocytes and spermatids and contained large unidentified multinucleated cells, possibly abnormal testicular oocytes (Figure 5D).

**Figure 6:**
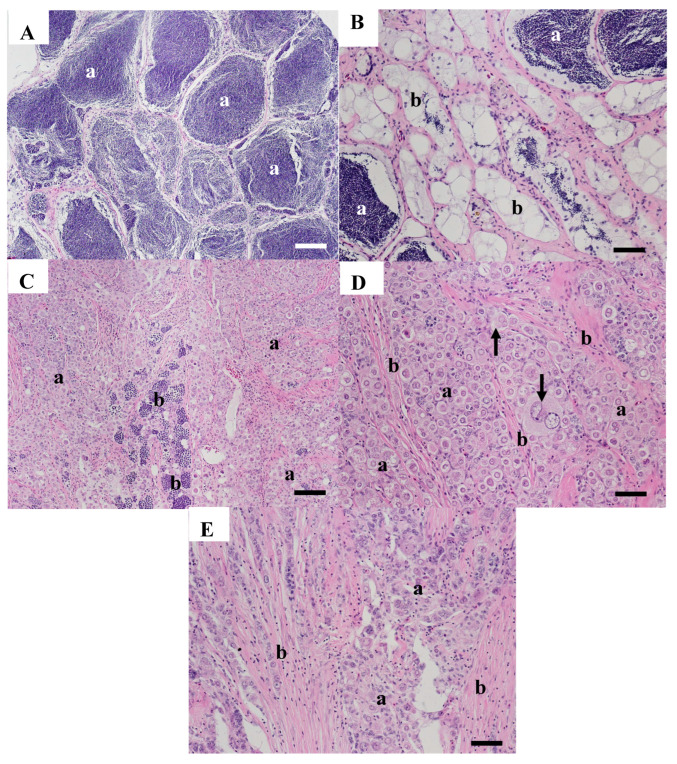
Microscopic observations of common carp WBCC-46 collected at Willow Beach in 2007. (**A**) Section of normal tissue with tubules containing sperm (a). Scale bar equals 100 µm. (**B**) Section with some tubules containing sperm (a). Other tubules have large vacuolated cells (b). Scale bar equals 50 µm. (**C**) Section of seminoma with sheets of neoplastic cells (a) around a focus of spermatocytes (b). Scale bar equals 100 µm. (**D**) Neoplastic cells (a) that varied in size were separated by fibrous tissue (b), and some were multinucleate (arrows). Scale bar equals 50 µm. (**E**). Area containing neoplastic germinal cells (a) and bundles of fibrous connective tissue (b). Scale bar equals 50 µm. H&E stain.

Neoplastic lesions were observed in the testes of three carp collected in 2007. A seminoma was present in testicular tissue from WBCC-46. There were areas of normal testicular tissue containing tubules filled with sperm (Figure 6A). Other areas had tubular structures lined with large vacuolated Sertoli cells. Tubules within these areas contained varying amounts of sperm within the lumen (Figure 6B). There were sheets of neoplastic spermatogonia-like cells, in some areas surrounding cysts of spermatogenic cells (Figure 6C). Neoplastic cells were pleomorphic, varied in size, and some had multiple nuclei. Sheets of neoplastic cells were separated by fibrous connective tissue (Figure 6D). Other areas had increased fibrosis interspersed with neoplastic germ cells (Figure 6E).

**Figure 7:**
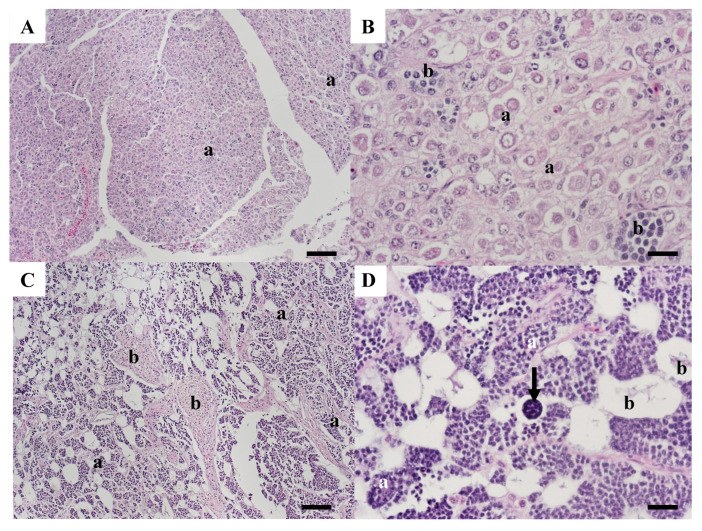
Microscopic pathology of common carp WBCC-50 testis collected at Willow Grove in 2007. (**A**) Areas of the testis contained sheets of neoplastic germ cells (a). Scale bar equals 100 µm. (**B**) These areas of the tumor had primarily neoplastic germ cells of varying size (a) with nests of spermatocytes (b). Scale bar equals 50 µm. (**C**) Other areas of the tumors contained primarily spermatogenic cells (a) and large testicular macrophage aggregates (b). Scale bar equals 100 µm. (**D**) These areas contained clusters of spermatogenic cells (a) and large vacuolated areas, which often contained debris (b) and some large, multicellular structures (arrow). Scale bar equals 50 µm. H&E stain.

Sections from WBCC-50 contained areas with sheets of neoplastic cells typical of a seminoma with no evidence of normal tubular structure (Figure 7A). Cells were pleomorphic, varied in size, and included large neoplastic germ cells with prominent nuclei and sometimes multiple nucleoli, as well as clusters of smaller, developing germ cells (Figure 7B). Other areas contained large MAs and small, dark-staining cells arranged around anastomosing spaces more consistent with an intratubular spermatocytic seminoma (Figure 7C). These cells included spermatocytes and spermatids. Membrane-bound vacuolated areas often contained cellular debris and sometimes multicellular structures (Figure 7D).

**Figure 8:**
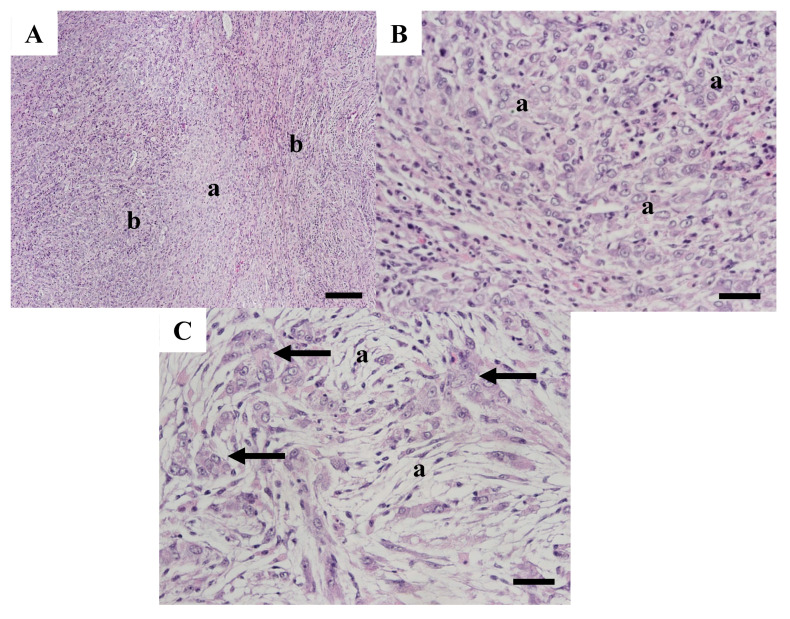
Testicular tissue from common carp WBCC-57 collected at the Willow Beach site in 2007. (**A**) Tissue was composed of sheets of neoplastic cells with areas of pale, primarily spermatogenic cells (a) interspersed with areas containing a mixture of spermatogenic and stromal cells (b). Scale bar equals 100 µm. (**B**) Some areas had primarily spermatogenic cells (a). Scale bar equals 50 µm. (**C**) Other areas contained large germ cells (arrows) interspersed with vacuolated spindle-shaped stromal cells (a). Scale bar equals 50 µm. H & E stain.

Testis tissue from WBCC-57 was more disorganized than tissues from the other carp testes and contained features of a mixed germ cell–stromal tumor. Tissue primarily contained sheets of neoplastic cells with no normal tubular formation (Figure 8A). The paler areas contained an increased number of germ cells (Figure 8B), while other areas contained large neoplastic germ cells interspersed with spindle-shaped neoplastic stromal cells with vacuolated cytoplasm (Figure 8C).

## 4. Discussion

Collections of adult carp have occurred at the WB site over many years—2003, 2007–2008, and 2010 [21,22,23]. Although testes were collected for histopathology in 2003 and 2007–2008, macroscopically abnormal testes were only documented during the 2007–2008 collections, and testicular tissue was only preserved for histopathology from a subsample of these fish. This was also the only sampling time with verified neoplasms. A number of other testicular abnormalities were observed in carp from WB in the other collections, including intersex and the presence of testicular MA. In fish, MAs or focal accumulations of pigmented macrophages, particularly splenic MA, have been identified as nonspecific indicators of environmental stress, including chemical exposure [25,26,27,28]. These pigmented macrophage aggregates, containing ceroid-lipofuscin deposits, as observed in carp testes, are derived from oxidized lipids or lipoproteins and hence are indicative of oxidative stress. Numerous large MAs were documented during the microscopic evaluation of testes from all sampling periods. Testicular MAs have been observed in numerous fish species, and increased size and number are suggested as a biomarker of environmental stress [20,29]. In red mullet (*Mullus barbatus*), a correlation between increased testicular MAs and high levels of organochlorines and alkylphenols [30] was observed. Other studies have suggested an association between testicular MAs and wastewater contaminants [31,32] or organic pollutants [33]. While chemical exposure is associated with an increased number and size of MA, age can also be an important cofactor in other tissues [25,28,34].

Some of the other testicular abnormalities observed in this study have been previously described in carp or carp hybrids, whereas others have not. Sertoli cells are somatic cells that support and regulate spermatogenesis. Fish have a cystic-type arrangement of gametogenesis (as opposed to a non-cystic type in mammalian species), and Sertoli cells retain the ability to proliferate in adults [35]. Within normal fish testes, Sertoli cell proliferation occurs predominantly during the period that germ cells are mitotically active [36]. Sertoli cells, located within the seminiferous epithelium, are known to have intrinsic plasticity, and two modes of proliferation occur in fish and amphibians. In the first mode, regulated by follicle-stimulating hormone (FSH), thyroid hormone, estrogens, and insulin-like growth factor, Sertoli cells proliferate to form new spermatogenic cysts. In the second mode, regulated by FSH, androgens, and progestins, these cells proliferate in existing cysts [37]. Consequently, the formation of tubules lined primarily with the putative Sertoli cells may be due to exposure to endocrine-disrupting chemicals. The cells we observed were highly vacuolated, most likely with lipid accumulation. Some of these cells were observed to have yellowish-brown pigment, believed to be ceroid/lipofuscin granules, as a consequence of lipid oxidation. There have been a few observations of abnormal Sertoli cell proliferation in fish in other studies. A testicular degenerative condition of unknown etiology, with an abnormal proliferation of Sertoli cells, was reported in a wild population of carp in Tasmania [38]. Yamaguchi et al. [39] described hypertrophied and vacuolated Sertoli cells in catfish *Pungasianodon hypophthalmus* and disturbed testis development associated with concentrations of the heavy metals arsenic, lead, molybdenum, and rubidium measured in the liver. Sertoli cell proliferations were noted in carp–goldfish hybrids from the Great Lakes. As in the current study, these Sertoli cells contained lipid droplets, and MAs were commonly associated with these proliferations [17].

Testicular tumors are recognized as difficult to diagnose due to their diversity and include germ cell (seminoma, spermatocytic seminoma, yolk sac, mixed) and sex cord—stromal (Leydig cell, Sertoli cell) neoplasms. They may have different growth patterns—diffuse and intratubular. Sertoli cell tumors, in particular, show a great deal of variation [40]. Both germ cell and stromal tumors have previously been reported in carp or hybrids, and the neoplasms observed in this study had characteristics of seminoma, spermatogenic seminoma, and mixed germ cell–stromal tumors. In mammals, all germ cell tumors arise from a neoplastic precursor cell that can differentiate into different pathways and include seminoma, nonseminomatous, and mixed tumors [41]. In humans, testicular germ cell cancers include seminomas (the most frequent malignant neoplasm in young men) and non-seminomas, both of which are most common in young men, and spermatocytic seminoma, which occurs most often in older men [42]. Seminomas are part of the testicular dysgenesis syndrome, an increasing reproductive issue of young men, that also includes declining semen quality, reduced serum testosterone concentrations, undescended testis, and hypospadias [43]. It has been suggested that exposure to environmental endocrine disruptors during early development is a risk factor [43,44].

Although a specific cause of carp testicular abnormalities described here is not known, a number of risk factors were identified. Fish age is an important factor in the occurrence of fish neoplasms, and increases in tumor prevalence with age have been reported in numerous studies [45,46,47]. For instance, English sole (*Parophyrs vetulus*) from contaminated sites in Puget Sound (Washington, USA) had a nearly 40% higher chance of having hepatic neoplasms for each additional year of age [48]. It was suggested that an important factor in neoplastic disease in koi carp is their longevity [15]. Carp were not aged in the 2007–2008 study; however, those aged in 2003 document an old population, varying in age from 35 to 54 years [21].

Another important factor could be the colder temperature and different seasonal thermal profile at WB when compared to the other sites in the watershed [22,23], which may influence fish growth and reproduction. Spawning of wild carp is influenced by seasonal water temperature cycles and is reported to begin when temperatures reach 15–17 °C [49,50]. Water temperatures measured during the 2007–2008 study documented little change in temperature seasonally (amplitude approximately 1.5 °C), and the highest temperature barely reached that required for spawning [23].

The high concentrations of polychlorinated biphenyls (PCBs) accumulated in male carp at WB may also be a contributing factor [21,23]. Male carp collected from this site in 2003 had the highest mean total PCB whole body concentration (0.87 µg/g wet weight (ww)) when compared to 10 other sites sampled in the same time period throughout the Colorado River (<0.048–0.12 µg/g ww). Only one site, downstream of Phoenix, AR, had higher concentrations (1.20 µg/g ww) [21]. This site also had the highest mean total PCB concentrations in male carp (0.50 µg/g ww) when compared to male carp collected at three other sites (0.04–0.25 µg/g ww) in the Lake Mead National Recreational Area in 2007 [23]. The age and water temperature combination may contribute to higher PCB levels. The older age of WB fish enables a longer time for bioaccumulation, and PCB elimination is much slower when fish are acclimated to lower water temperatures [51]. Testicular cancer in humans has been increasing during the past decades, particularly in the USA and some European countries [52,53]. Testicular germ cell tumors (seminoma and non-seminoma) were significantly associated with estrogenic PCB plasma concentrations in a study conducted in Connecticut and Massachusetts, USA [54]. Further research on the association of PCB gonad concentrations could determine if the oxidative damage (MA) and Sertoli cell proliferations may be preneoplastic indicators for testicular neoplasms in carp.

The decrease in the mean whole body PCB concentrations between 2003 and 2007 suggests other contaminants could also play a role. Studies have associated testicular cancer with exposure to endocrine-disrupting chemicals, including pesticides and phthalates [55] and organochlorines/organohalogens [56]. Using the yeast estrogenicity screen assay, estrogenic activity at WB exceeded the long-term, environmentally safe criteria of 0.1–0.4 ng/L [57] by at least 7.8 times [22]. Further studies would be necessary to identify potential estrogenic emerging contaminants.

## 5. Conclusions

This study utilized testicular tissue collected during a study focused on identifying physiological and organ-level biomarkers in carp collected in 2007 at sites along multiple gradients of contaminant exposure within the Lake Mead National Recreation Area within the Colorado River watershed. Observations of visibly abnormal testes at the Willow Beach site led investigators to preserve pieces of abnormal and normal-appearing tissue from a subset of fish. Testicular tumors, including seminoma and mixed germ cell-stromal neoplasms, as well as numerous large MA, fibrosis, intersex, and Sertoli cell proliferation were observed microscopically. Examining archived histology slides from a study throughout the Colorado River that included the Willow Beach site allowed us to retrospectively compare testicular pathology over time. Although neoplasms were not observed in the earlier study, similar non-neoplastic (possibly pre-neoplastic) abnormalities were present. Testicular MA, indicators of chronic inflammation and oxidative tissue damage, and the proliferative response of Sertoli cells may be early signs of gonadal carcinogenesis. Age, water temperature, PCB exposure, and other environmental stressors may all play a role in the development of the preneoplastic and neoplastic changes observed.

## Figures and Tables

**Table 1:** Testicular tissues of common carp from Willow Beach examined microscopically.

Year Collected	Length Millimeters	Age Years	Sample Number	Gonad Stage	Observations
2003	467	35	320-2	4	MA ^1^
2003	500	42	320-3	3	Normal
2003	510	53	320-6	3	MA, Sertoli cell proliferation
2003	575	47	320-7	3	Normal
2003	525	50	320-9	3	MA
2003	550	39	320-10	3	MA
2003	465	38	320-11	3	MA
2003	590	37	320-12	4	Sertoli cell proliferation
2003	468	44	320-15	3	Normal
2007	438	ND ^2^	WBCC-46	3	MA, Sertoli cell proliferation, neoplasia
2007	521	ND	WBCC-50	Unknown	MA, neoplasia
2007	442	ND	WBCC-52	3	MA, intersex
2007	471	ND	WBCC-57	Unknown	Neoplasia
2007	442	ND	WBCC-66	3	MA, Sertoli cell proliferation

^1^ MA = numerous testicular pigmented macrophage aggregates; ^2^ ND = no data.

## Data Availability

The new data created for this manuscript were the histopathological descriptions of the tumors and other abnormalities, and they are all found within the manuscript.
